# Efficiency of fine scale and spatial regression in modelling associations between healthcare service spatial accessibility and their utilization

**DOI:** 10.1186/s12942-021-00276-y

**Published:** 2021-05-19

**Authors:** Fei Gao, Clara languille, Khalil karzazi, Mélanie Guhl, Baptiste Boukebous, Séverine Deguen

**Affiliations:** 1 HESP, 35000 Rennes, France; 2 Recherche en Pharmaco-Épidémiologie Et Recours Aux Soins, L’équipe REPERES, UPRES EA-7449, Rennes, France; 3 Department of Social Epidemiology, INSERM, Sorbonne Université, Institut Pierre Louis D’Épidémiologie Et de Santé Publique, IPLESP, 75012 Paris, France; 4 Univ Rennes, Ensai, 35000 Rennes, France; 5 ECAMO, UMR1153, CRESS, INSERM, Paris, France; 6 Hoptial Bichât /Beaujon, APHP, Paris, France; 7 Department of Quantitative Methods for Public Health, EHESP School of Public Health, Avenue du Professeur Léon Bernard, 35043 Rennes, France

**Keywords:** Spatial accessibility, Utilization of healthcare, Hospital care, Primary care, Length of stay, Administrative data utilization, OLS, GWR, SAR

## Abstract

**Background:**

Healthcare accessibility, a key public health issue, includes potential (spatial accessibility) and realized access (healthcare utilization) dimensions. Moreover, the assessment of healthcare service potential access and utilization should take into account the care provided by primary and secondary services. Previous studies on the relationship between healthcare spatial accessibility and utilization often used conventional statistical methods without addressing the scale effect and spatial processes. This study investigated the impact of spatial accessibility to primary and secondary healthcare services on length of hospital stay (LOS), and the efficiency of using a geospatial approach to model this relationship.

**Methods:**

This study focused on the ≥ 75-year-old population of the Nord administrative region of France. Inpatient hospital spatial accessibility was computed with the E2SFCA method, and then the LOS was calculated from the French national hospital activity and patient discharge database. Ordinary least squares (OLS), spatial autoregressive (SAR), and geographically weighted regression (GWR) were used to analyse the relationship between LOS and spatial accessibility to inpatient hospital care and to three primary care service types (general practitioners, physiotherapists, and home-visiting nurses). Each model performance was assessed with measures of goodness of fit. Spatial statistical methods to reduce or eliminate spatial autocorrelation in the residuals were also explored.

**Results:**

GWR performed best (highest R^2^ and lowest Akaike information criterion). Depending on global model (OLS and SAR), LOS was negatively associated with spatial accessibility to general practitioners and physiotherapists. GWR highlighted local patterns of spatial variation in LOS estimates. The distribution of areas in which LOS was positively or negatively associated with spatial accessibility varied when considering accessibility to general practitioners and physiotherapists.

**Conclusions:**

Our findings suggest that spatial regressions could be useful for analysing the relationship between healthcare spatial accessibility and utilization. In our case study, hospitalization of elderly people was shorter in areas with better accessibility to general practitioners and physiotherapists. This may be related to the presence of effective community healthcare services. GWR performed better than LOS and SAR. The identification by GWR of how these relationships vary spatially could bring important information for public healthcare policies, hospital decision-making, and healthcare resource allocation.

## Background

Accessibility to healthcare is widely recognized as a critical issue in public health and plays a fundamental role in improving health outcomes [1–11]. One of the most common conceptualizations of healthcare accessibility was developed by Andersen who characterized access along several dimensions, including potential and realized access [12, 13]. Potential access, also known as spatial access, refers to the spatial distribution of healthcare facilities, and the possibility to reach healthcare activity locations or services from a given position or by an individual [14–18]. Potential access represents only one dimension of healthcare accessibility because the presence of nearby healthcare facilities may not result in realized access [19–21], and individuals who can access such healthcare services may choose not to use them [22]. Realized access refers to the actual utilization of healthcare services [23] and the real interaction with the healthcare system [24]. In the last two decades, much geographic research on healthcare access has focused on the potential access to services by measuring the spatial accessibility of medical services, rather than on the realized access (i.e. the utilization of health services) [25, 26]. However, measuring both potential and realized access is mandatory in order to address the diversity of demands by providers, patients as well as policy makers. Moreover, healthcare service utilization could be strongly influenced by the potential access [27–29]. Yet, less research effort has been dedicated to understand how potential accessibility affects healthcare utilization [26].

The assessment of the spatial access to healthcare services and their utilization should also take into account the care provided by the various facility types: ambulatory/home care, which is the basis of primary care in many countries [30, 31], and inpatient hospital care, also called secondary care, including both acute and long-term care hospitals [32–35]. Spatial access to and utilization of these two healthcare types are closely linked. Previous research findings suggest that patients who face geographic barriers to primary care may use more frequently hospital services [36–39]. This is the case of patients living in medically underserved areas [38, 40, 41]. Moreover, the ability of primary healthcare services to take care of discharged patients has a significant impact on the hospitalization length [33, 42, 43]. Length of hospital stay (LOS) is a classical indicator of hospital care utilization, and is interpreted as a measure of the healthcare supply and treatment efficiency [33, 44–46]. Investigating the effect of spatial accessibility to primary and secondary care services on LOS is important to better understand the complex links between healthcare spatial accessibility and utilization, and between hospital and ambulatory/home healthcare [47–49]. This knowledge is crucial for efficient resource planning and allocation [50–52].

Finally, many previous studies on the relationship between healthcare spatial accessibility and utilization used conventional statistical methods without addressing the scale effect and spatial processes [37, 39, 53–55]. However, this relationship relies on spatial data and is influenced by the associated spatial effects [29, 56–58].

Healthcare utilization data have already been used at the country, regional, district or zip code levels to study the geographical and temporal dynamics of epidemics and their correlation with and environmental factors [59–62]. Moreover, it has been suggested that for public health planning, analyses at a finer spatial scale are more efficient to better identify and target critical areas that require intervention scaling up [63–65]. Indeed, such analyses could significantly increase the accuracy of the estimates about future healthcare utilization demands (e.g. number of hospital beds) [66, 67], and could capture the heterogeneity of spatial accessibility [68]. However, few studies have addressed the scale effect by comparing quantitatively the efficiency of fine and large spatial scale analyses, especially when analysing the relationship between healthcare spatial accessibility and utilization.

Moreover, spatial data exhibit spatial non-stationarity and spatial autocorrelation [69]. Spatial autocorrelation can be defined as the self-similarity of nearby observations [70]. Therefore, autocorrelated residuals may increase the level of uncertainty of the regression coefficients, and usually lead to larger prediction intervals [71]. Spatial autocorrelation has been described for geographical and spatial economic phenomena and may be readily dealt using spatial regression models [72, 73]. The residual spatial autocorrelation issue is commonly addressed using the spatial autoregressive (SAR) model that takes into account spatial dependency. Non-stationarity is another spatial issue. Most studies suggested generalized results across all locations [29, 37, 39, 42, 55, 74], and ignored how the relationship between healthcare spatial accessibility and utilization may take different forms in different places. Spatial non-stationarity occurs when the strength and direction of the relation in one place does not apply in another. Compared with global models in which results are assumed to be stationary and could be generalized to all locations, the outcomes of local models are location-specific. A generalized conclusion does not fully take into account specific local situations and may mislead public policy-makers [75–78]. Geographically weighted regression (GWR) allows such analysis and can help to identify relationships that remain hidden when using global models, such as ordinary least squares (OLS) and SAR. Using both SAR and GWR allows considering the global and local spatial scales, and can help to identify all factors, their impact on dependent variables, and their interdependence [79]. Moreover, to the best of our knowledge, no study has compared the efficiency of a traditional regression (OLS) with a geospatial approach (SAR and GWR) for modelling the relationship of potential and realized healthcare access.

Therefore, the main objective of this work was to assess the efficiency of fine spatial scale analyses for investigating the impact of spatial accessibility to primary and secondary healthcare services on the length of hospital stay, relative to geospatial variables. This study had two specific objectives: i) to assess the efficiency of a fine spatial scale analysis to identify hidden factors in the relationship between LOS and healthcare spatial accessibility; and ii) to explain the spatial variability of this association by comparing traditional (OLS) and geospatial modelling approaches (SAR and GWR), thus providing a case study on the application of geospatial techniques for the analysis of this relationship.

## Material and methods

### Study setting and population

This study was carried out in the Nord administrative region that is located in the north of France, with a surface area of 5743 km^2^ and a population density of 456 inhabitants per km^2^. Several primary care spatial accessibility indicators are available for this department [80–83]. Moreover, it has been shown that edge effects lead to minor spatial accessibility variations in this area [84]. The study focused on the ≥ 75-year-old group of the population of this region, because many different healthcare resources, such as hospital facilities and primary care professionals, are involved in their management. Moreover, as their recovery period after a hospital stay is often longer, their length of stay could be more influenced by the capacity of the primary healthcare services to manage them [47–49].

### Statistical unit

As the first objective was to assess the efficiency of a fine spatial scale analysis, this study was carried out first at the French Geographic Code unit (FGC) level and then at the sub-municipal French census block group level (known as IRIS: “Ilots Regroupés pour l’Information Statistique”). The FGC statistical unit is defined by the French national discharge database, and is approximately equivalent to the municipality postal code. The Nord administrative region is divided into 240 FGCs with a population ranging from 1,000 to 227,000 inhabitants per FGC. IRIS is defined by the French National Institute of Statistics and Economic Studies (INSEE) [85], and is the smallest infra-urban level for which complete French census data are available. There are 1,346 IRIS units in the Nord administrative region with a population ranging from 6 to 5,414 inhabitants per IRIS. A FGC can include 1 to 110 IRIS units, but one IRIS only belongs to one FGC.

### Data sources

Multiple data sources were combined for the present study.

#### Index of spatial accessibility (ISA)

**Fig. 1:**
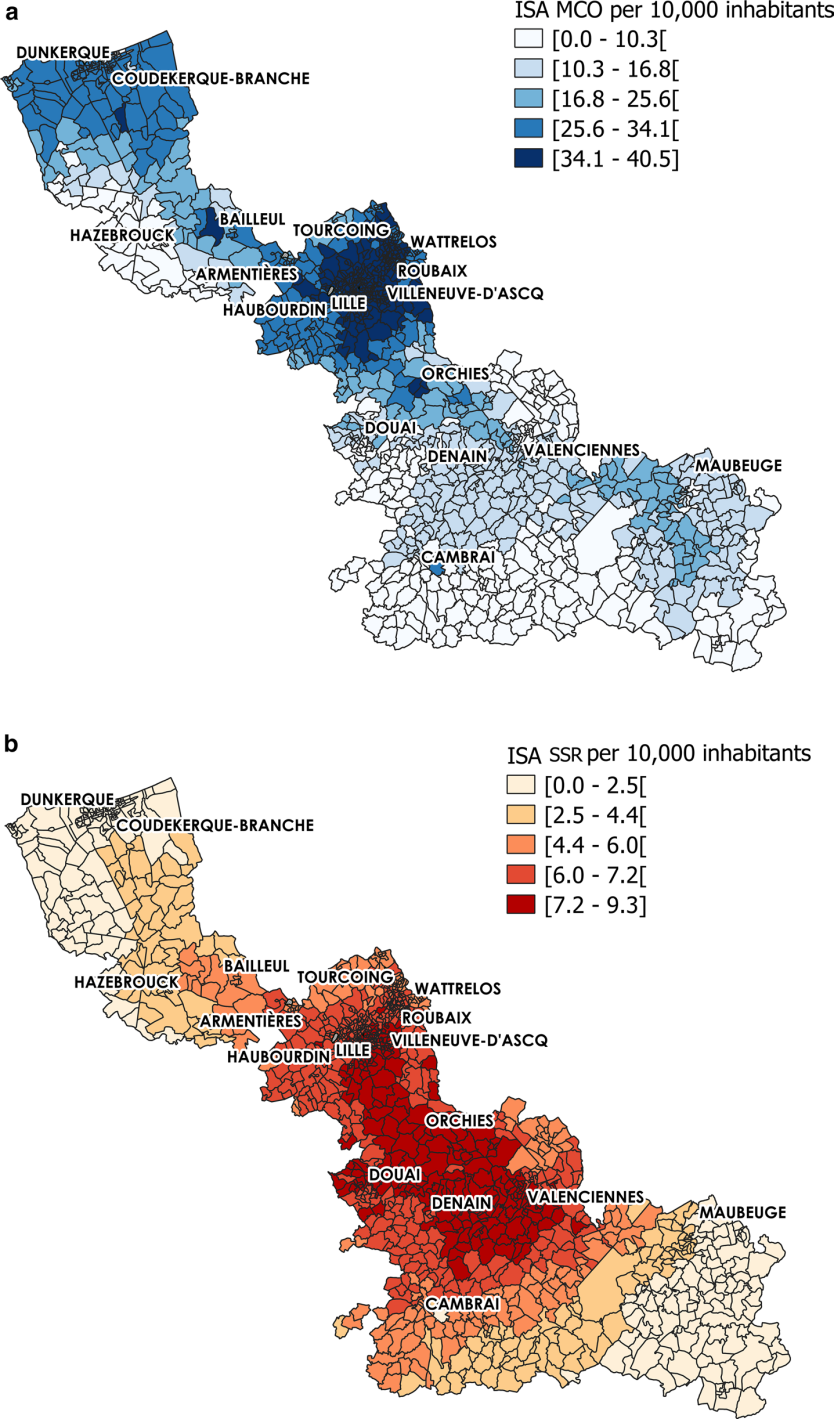
Index of Spatial Accessibility distribution at the IRIS level. **a** Index of Spatial Accessibility (ISA) for Medical, Surgical and Obstetrics (MCO) and **b** Postoperative and Rehabilitation Care (SSR) centres. For each map, the Nord administrative region is represented using a graduated colour scale to highlight the ISA score variability among IRIS units

ISA is a previously developed indicator to measure spatial accessibility to hospital care (inpatients). Based on the enhanced two-step floating catchment area (E2SFCA) method, ISA takes into account the number of beds in Medical, Surgical and Obstetrics facilities (Médecine, Chirurgie, Obstétrique: MCO) and Postoperative and Rehabilitation Care facilities (Soins de Suite et de Réadaptation: SSR) facilities, the car travel time, and the population distribution. ISA construction steps have been previously described [83, 86]. It is a special form of physician-to-population ratio, expressed as the number (N) of beds per 10,000 inhabitants. Higher scores indicate higher accessibility. This indicator was initially developed at the census block (IRIS) scale, and was then summarized at the French Geographic Code (FGC) scale for MCO and SSR facilities. On average, there are 22.69 beds in MCO and 5.49 beds in SSR for 10,000 inhabitants in the Nord administrative region. ISA spatial distribution revealed important variations within IRIS units (Fig. 1). Specifically, the highest ISA values for MCO were observed in urban areas located in the northern part of the studied territory and also in the centre. Conversely, the lowest values were observed mostly in the southern part. The highest ISA scores for SSR were concentrated in the middle part of the region, whereas access was lower in the North and South.

#### Length of stay

The Length Of Stay (LOS) was defined as the mean hospital stay length of elderly people (≥ 75 years of age) relative to the total ≥ 75-year-old population in a given FCG (Eq. 1).

1 $${LOS}_{i}=\sum_{g>75}\frac{{Average\, length\, of\, stay}_{gi}}{{P}_{gi}}$$

where *gi* represents the three groups of ≥ 75-year-old people (75–84, 85–94 and > 95 years) for a given spatial unit i, and *P*_*gi*_ the corresponding total population for that age group. The numerator represents the average length of stay of each age group, standardized to the total population for that age group (inpatients or not) in the denominator. The mean LOS was chosen instead of the median LOS in order not to underweight extreme LOS values. Indeed, in this context of healthcare resource allocation, extremely long LOS do exist and need to be taken into account.

The LOS was calculated from the French national discharge database that collects hospital activity and patient discharge data [87–90] using MCO and SSR inpatient care data for the year 2014. The number of ≥ 75-year-old people in the Nord administrative region was obtained from the 2016 French national Census [85].

#### Localized potential accessibility (APL)

The spatial accessibility to primary (ambulatory/home) care services was described using the Localized Potential Accessibility (Accessibilité potentielle localisée: APL) database [80]. Based on the E2SFCA method, APL indices are available at the national level but only for eight types of healthcare professionals: general practitioners (GPs), physiotherapists, home-visiting nurses, paediatricians, dental surgeons, midwives, gynaecologists, and ophthalmologists. For this study, the APL indices for GPs, physiotherapists, and home-visiting nurses were used because these three types of health professionals could contribute substantially to the primary care services to older adults. Furthermore, the healthcare provided by them might interact with inpatient hospital care. APL indices are available at the FGC level and are expressed for 10,000 inhabitants.

#### Socioeconomic variables

Although this was not the principal aim of the study, several neighbouring socioeconomic variables were introduced because they also could affect healthcare utilization behaviours, as previously reported [36, 91–94]. From the initial database of economic variables [85] that might influence care utilization, data pre-processing was performed using the variance inflation factor (VIF) to identify the presence of multicollinearity among covariates. VIF values lower than 10 are considered acceptable [95, 96]. The final variables included in the model for further analysis and the scale at which they are available are summarized in Table 1.

**Table 1:** Variable description

Variables	Description	Available	Care level
Dependent variable LOS_MCO/ LOS_SSR	The mean hospital stay length in MCO or SSR of elderly people (≥ 75 years of age) relative to the total ≥ 75-year-old population	FGC scale	Secondary
Independent variables ISA_MCO/ ISA_SSR APL_GPs APL_Nurses APL_Physiotherapists	Index of spatial accessibility to MCO and SSR facilities Localized Potential Accessibility to general practitioners Localized Potential Accessibility to home-visiting nurses Localized Potential Accessibility to physiotherapists	IRIS scale FGC scale FGC scale FGC scale	Secondary Primary Primary Primary
Economic variables Non-Owner Precarious	Percentage of inhabitants who do not own their main property Percentage of inhabitants with a precarious situation	IRIS scale IRIS scale	– –

### Methodology

#### Ordinary least squares model

Multiple linear regression models are frequently used for predictive and explanatory analyses and the Ordinary Least Squares (OLS) method allows estimating the best fit. An OLS model treats data as independent, assuming that any variation due to spatial variability within the area units is not captured by the method.

In our study, the OLS model was expressed by the following function:

2 $$ln\left({LOS}^{*}\right)={\beta }_{0}+ {\beta }_{1}.ln\left({ISA}^{*}\right)+{\beta }_{2}.precarious +{\beta }_{3}.nonOwner+\sum {\beta }_{n}.\mathrm{ln}\left({APL}_{n}\right)+ \varepsilon ;with \varepsilon \approx iid\left(0;{\sigma }^{2}\right)$$

where:

- signifies that the LOS for MCO (with the corresponding ISA as independent variable) and for SSR (with the corresponding ISA as independent variable) were modelled separately.
- *n* defines the number of different types of primary healthcare professionals considered in the analysis (*n* = 3: GPs, physiotherapists, and home-visiting nurses).
- The other covariates, namely Precarious, Non-Owner and APL, are defined in Table 1.

#### Spatial non-stationarity and spatial autocorrelation: towards spatial statistical methods

Traditional regression models, such as OLS, consider only the stationary relationship between dependent and independent variables, ignoring the spatial autocorrelation and non-stationarity [97]. However, spatial effects, such as spatial heterogeneity and spatial autocorrelation, can affect the accuracy of OLS estimates by increasing the regression errors and uncertainty [98]. Therefore, two alternative spatial modelling methods were assessed to overcome these limitatons.

##### Spatial AutoRegressive model

3 $$ln\left({{LOS}^{*}}_{i}\right)=\rho \sum {w}_{ij}ln\left({{LOS}^{*}}_{j}\right)+{\beta }_{0}+ {\beta }_{1}.ln\left({{ISA}^{*}}_{i}\right)+{\beta }_{2}.{precarious }_{i}+{\beta }_{3}.{nonOwner}_{i}+\sum {\beta }_{n}.\mathrm{ln}{\left({APL}_{n}\right)}_{i}+ {\varepsilon }_{i};with {\varepsilon }_{i}\approx iid\left(0;{\sigma }^{2}\right).$$

The Spatial AutoRegressive (SAR) model takes into account spatial dependency and addresses the spatial autocorrelation problem by adding a spatial lag term, $\rho WY$, in Eq. 2 that defined the OLS model. The SAR model is based on the assumption that a dependent variable at a location is affected by the dependent variable of neighbouring locations in addition to the effects of independent variables [99]. The values of the dependent variable of neighbouring geographic units are averaged and become a term on the independent side of the equation. The model equation is as follows: where:

- $\rho$ is the spatial autoregressive coefficient. It quantifies the effect of neighbour observations of $ln\left({{LOS}^{*}}_{j}\right)$ and the direction of that effect [100].
- $ln\left({{LOS}^{*}}_{i}\right)$ is partly explained by the values taken by $ln\left({LOS}^{*}\right)$ in the neighbouring geographic units.
- $\sum {w}_{ij}$ is the spatial lag for the n x n weight matrix $W$, and represents the proximity between each pair of geographic units $\left(i,j\right)$.
- The spatialreg R package was used for this model [101]. To construct the spatial weight matrix, both rook and queen contiguity weights were analysed and the rook contiguity, which defines adjacent polygons as those sharing edges, was selected due to its slightly higher R^2^.

##### Geographically weighted regression model

Global models, such as OLS and SAR, may mask potential spatial non-stationarity. To explore LOS spatial non-stationarity, the GWR model was implemented. This model considers that coefficients vary across space, by estimating different relationships between the dependent and independent variables for each geographic location. The purpose of GWR is to embed the geographical location into parameters based on a traditional regression, in order to establish a spatial-weighting matrix and run a local weighted regression for each area unit to allow the analysis of the spatial variation and related driving factors of the research object at a specific scale. Regression coefficients are then defined as local coefficients. Therefore, the GWR model is more suitable for studying the spatial heterogeneity and local effects, and truly depicts the local influence of independent variables on dependent variables. The model is described by the following equation:

4 $$ln({{LOS}^{*}}_{i})={\beta }_{0}\left({u}_{i},{v}_{i}\right)+ {\beta }_{1}\left({u}_{i},{v}_{i}\right).ln\left({ISA}^{*}\right)+{\beta }_{2}\left({u}_{i},{v}_{i}\right).precarious +{\beta }_{3}\left({u}_{i},{v}_{i}\right).nonOwner+\sum {\beta }_{n}\left({u}_{i},{v}_{i}\right).\mathrm{ln}\left({APL}_{n}\right)+ \varepsilon ;with \varepsilon \approx iid\left(0;{\sigma }^{2}\right)$$

where:

- ${(u}_{i},{v}_{i})$ denotes the coordinates of each data point where local regressions are calculated.
- At location $i,$ $ln({{LOS}^{*}}_{i})$ is the local dependent variable; ${\beta }_{0}$ and ${\beta }_{k}$ represent the local estimate intercept and coefficient of factor $k,$ respectively [96, 102, 103].

At each regression area unit, local coefficients are estimated according to a spatial weighting scheme, characterized by two elements: (1) the neighbouring window of the area unit, and (2) the distance decay function used to calculate the spatial weights for each area unit in the window.

The spatial weighting function employed in a GWR model assumes that neighbouring area units in a window have more similar characteristics compared with those distant from each other. Thus, the area unit parameters ${\beta }_{k}\left({u}_{i},{v}_{i}\right)$ are more strongly influenced by closer observations than by units further away. The neighbouring window of each area unit can be determined using fixed or adaptive kernel types. The fixed type selects an optimal global bandwidth for the whole area; all area units that fall within the bandwidth are included in the regression. The adaptive type adjusts the size of the spatial window by choosing a specified number of nearest neighbours. The kernel function modifies the weights given to each neighbouring unit according to its distance from the regression unit. In this study, the adaptive kernel approach was used because the geographical unit size in the study area varied greatly, and therefore the fixed bandwidth approach appeared irrelevant. The Gaussian function was selected to minimize the Akaike information criterion (AIC). The spatial weight matrix was constructed with the rook contiguity method and the GWR models were implemented using the gwr R package [104].

#### Model assessment and comparison

##### Global Moran’s I

The Moran test was first employed to assess the global autocorrelation of the dependent variable LOS. The Global Moran’s I index was estimated to quantify the level of spatial autocorrelation (or clustering) of LOS. Moran’s I statistic values range from − 1 to + 1, depending on the degree and direction of the association. Values significantly close to 1 indicate positive spatial autocorrelation, and values significantly close to − 1 indicate negative spatial autocorrelation. A number approaching 0 indicates the absence or a small spatial autocorrelation level, which means that data have a random spatial relationship.

##### Monte-Carlo test for the GWR model

It is important to note that the GWR model does not assume that relationships vary across space, but can identify whether they do or not. If the relationships do not vary across space, the global model could be appropriate for the data. Therefore, the Monte-Carlo test is used to evaluate the spatial variability of individual parameters or coefficients in a GWR model [102, 105–107]. The main idea of the Monte-Carlo test is that in the absence of relevant spatial phenomena, the geographical coordinates of the observations could be permuted randomly for a certain number of times. Consequently, the variance remains unchanged and the p-values can be estimated.

##### Measures of goodness of fit

Two goodness of fit measures were implemented to compare the OLS, SAR and GWR approaches: the adjusted coefficient of determination (adjusted R^2^) and the AIC [108, 109]. R^2^, one of the most popular measures, indicates the variance proportion of a dependent variable that is explained by an independent variable in a regression model. The model performance is proportional to the R^2^ value. AIC estimates the robustness of each model, relative to each of the other models. The best model is the one with the lowest AIC index value.

##### Analysis of residuals: Moran’s I and Local Indicators of Spatial Association

A posteriori, the global Moran’s I and local indicators of spatial association (LISA) were used to determine whether the three models (OLS, SAR and GWR) could efficiently eliminate spatial autocorrelation in the residuals of their estimates.

The LISA indicator, also known as Local Moran’s I, was developed by Anselin in 2005 [110]. While the global Moran’s I statistic shows whether overall, spatial autocorrelation is present or not, LISA identifies the presence of spatial autocorrelation clusters at specific locations. Four types of local spatial associations between an observation point and its neighbours can be detected: High-High (HH), Low-Low (LL), High-Low (HL) and Low–High (LH). A first-order rook contiguity relationship was chosen as the spatial weight for the LISA clustering analysis. The Geoda 0.9.5 software was used for the LISA (Local Moran’s I) analyses [111].

#### Statistical strategy

The main objective was to assess the efficiency of fine spatial scale analyses.

First, the OLS regression model was used to examine the global linear relationship between LOS and spatial accessibility to inpatient hospital care and to the three types of primary care services (GPs, physiotherapists, and home-visiting nurses). This part of the study was carried out first at the FGC level and then at the IRIS level. The OLS model was considered as the reference model in this study.

Then, the efficiency of a geospatial modelling approach to enhance our understanding of the relationship between LOS and healthcare spatial accessibility was tested. After assessment of the spatial autocorrelation with the Global Moran’s I test, the SAR and GWR models were applied and compared with the results of the OLS model previously carried out. The performance of each model (OLS, SAR, and GWR) was assessed using measures of goodness of fit.

Finally, the spatial autocorrelation and spatial non-stationarity of the residuals of these models were assessed, and the efficacy of the spatial statistical methods to reduce or eliminate spatial autocorrelation in the residuals was also explored [112].

In summary, by using the OLS, SAR and GWR models, the LOS model can be expressed as follows:

OLS: Length of Hospital Stays = *F* (inpatient hospital spatial accessibility, primary care service spatial accessibility, Precarious, Non-Owner)

SAR: Length of Hospital Stays = *F* (Spatially Adjacent Neighbour’s length of hospital stays, inpatient hospital spatial accessibility, primary care service spatial accessibility, Precarious, Non-Owner)

GWR: Length of Hospital Stays = *f* (inpatient hospital spatial accessibility, primary care service spatial accessibility, Precarious, Non-Owner),

where *f* is the regression equation for each observation in a dataset, influenced or weighted to a greater degree by the variables of other observations nearer to it.

## Results

### LOS basic statistics

**Fig. 2:**
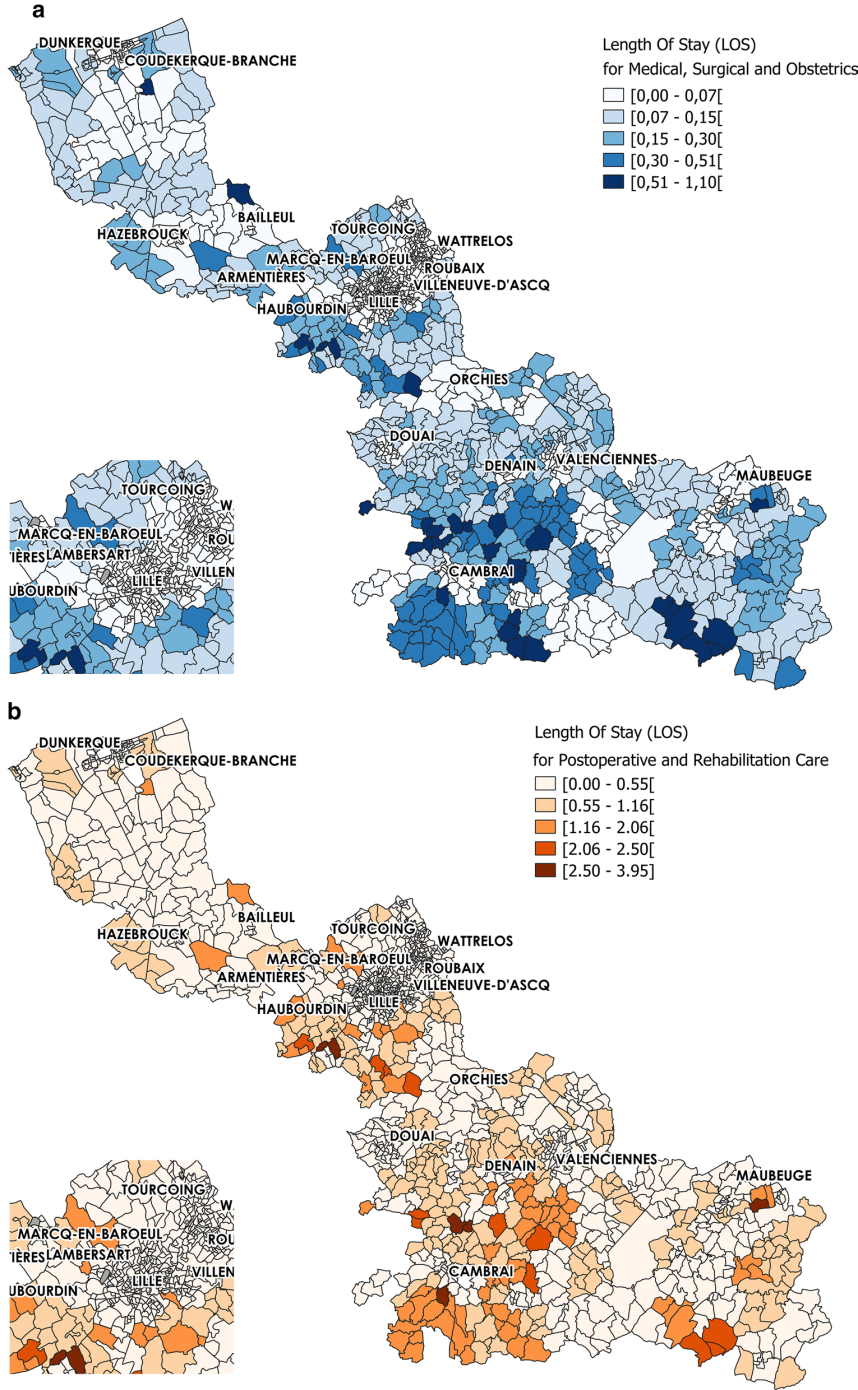
Length Of Stay spatial distribution for ≥ 75-year-old people at the IRIS level. **a** Length Of Stay in Medical, Surgical and Obstetrics and **b** Post-operative and Rehabilitation Care centres

Analysis of the LOS for the ≥ 75-year-old population in the Nord administrative region (Table 2) showed that in 2014, the mean LOS values were 0.26 and 0.85 for MCO and SSR, respectively. LOS was initially constructed at the FGC level, and then disaggregated at the IRIS level. The spatial variation was important, with standard deviations of 0.20 and 0.92 for MCO and SSR, respectively. The LOS_MCO and LOS_SSR score distribution highlighted a non-homogeneous repartition with higher values close to the border with other French regions, especially in the southern part (Fig. 2). Lower scores were observed from Dunkerque to Bailleul, and also around Lille, Roubaix, Orchies and Tourcoing.

**Table 2:** LOS of ≥ 75-year-old people in MCO and SSR facilities at the IRIS scale – Nord administrative region

N	Min	Mean (Sd*)	Max	25th	Median	75th
LOS_MCO						
1346	0.01	0.26 (0.20)	1.1	0.1	0.2	0.37
LOS_SSR						
1346	0.03	0.85 (0.92)	3.95	0.33	0.65	1.08

### Efficiency of the fine scale analysis

OLS regression analysis was used to compare the efficiency of the spatial analysis at the FGC and IRIS scales. For analyses at the FGC level, independent variables available at the IRIS level (ISA, Non-Owner and Precarious) were summarized by taking into account the population density. Conversely, for analyses at the IRIS level, variables available only at the FGC level (APL and LOS) were disaggregated by giving the same value to each IRIS included in the same FGC.

The OLS regression analysis (regression coefficient estimates and the corresponding p-values are in Table 3) showed that for LOS at MCO facilities, three variables were significant at the FGC level (p-value < 0.01): spatial accessibility to GPs (APL_GPs), percentage of inhabitants who do not own their main property (Non-Owner), and percentage of inhabitants with precarious employment (Precarious). APL_GPs and Non-Owner values were negatively correlated with LOS, suggesting that a better spatial accessibility to GPs might decrease LOS, and that hospital stays at MCO facilities tend to be shorter for elderly people living in disadvantaged area units where more people do not own their main property.

**Table 3:** OLS regression analysis of length of stay (LOS) at MCO and SSR facilities

	FGC			IRIS		Comparison	
Predictor variables	Coefficients	Std Error		Coefficients 0.161***	Std Error	β increase	Std Error decrease
LOS_MCO							
ISA_MCO	− 0.057	0.087		− 1.065**	0.037	184.64%	− 57.74%
APL_GPs	− 0.900***	0.201		0.302***	0.097	18.24%	− 52.12%
APL_Nurses	0.349	0.192		− 1.518***	0.096	− 13.48%	− 49.51%
APL_Physiotherapists	− 0.169	0.295		− 1.395***	0.165	799.48%	− 43.92%
Non-Owner	− 3.652***	0.425		− 0.507	0.117	− 61.80%	− 72.43%
Precarious	5.033**	1.739			0.403	− 110.08%	− 76.84%
Model assessment							
Adjusted R^2^	0.389			0.508			
AIC	528.716			3285.273			
S_SSR							
ISA_SSR	0.103		0.069	0.245***	0.036	136.72%	− 48.16%
APL_GPs	− 0.895***		0.172	− 1.367***	0.096	52.53%	− 47.61%
APL_Nurses	0.294		0.192	0.332***	0.09	12.81%	− 49.85%
APL_Physiotherapists	− 0.189		0.298	− 1.679***	0.165	787.36%	− 44.58%
Non-Owner	− 3.575***		0.424	− 1.209***	0.114	− 66.17%	− 73.01%
Precarious	5.452**		1.737	− 0.491	0.384	− 109.00%	− 77.91%
Model assessment							
Adjusted R^2^	0.352			0.485			
AIC	525.425			3255.05			

**p < 0.01; *** p < 0.001; Std Error: Standard deviation error

When the OLS model was run at the IRIS scale, all independent variables showed the same direction of association (positive or negative) with LOS as observed at the FCG level, but for the Precarious variable that was no longer significant at an α-risk level of 0.05. The number of variables showing significant relationships with LOS increased from three to five. Specifically, spatial accessibility to home-visiting nurses and physiotherapists, and to MCO facilities became significant. LOS was negatively correlated with APL_Physiotherapists and APL_GPs. However, the regression results at the IRIS level suggested that better spatial accessibility to home-visiting nurses corresponded to longer LOS. Furthermore, LOS was shorter for patients with easier access to a MCO facility (ISA_ MCO).

Similar results were obtained for the analysis of LOS at SSR facilities, with only two and five significant variables at the FGC and IRIS level, respectively. Among the significant variables at the IRIS level, only ISA_SSR displayed an opposite sign compared with the analysis for MCO facilities, showing longer LOS for patients with easier access to SSR facilities.

The adjusted R^2^ for the OLS regression analyses increased from 0.389 and 0.352 at the FGC level to 0.508 and 0.485 at the IRIS level for MCO and SSR facilities, respectively. The standard errors decreased accordingly (from 44 to 77% for all coefficients).

In the next steps of this study, the OLS model at the IRIS scale was considered as the reference model and was compared with the other two spatial models.

### Efficiency of the geospatial modelling approach

SAR and GWR analyses were carried out to address the issues of spatial autocorrelation and non-stationarity, respectively.

### Coefficient estimates

Table 4 summarizes the results obtained with the three different models. In the OLS regression model, LOS in MCO was negatively correlated with spatial accessibility to MCO facilities (ISA_MCO), spatial accessibility to GPs and physiotherapists (APL_GPs and APL_Physiotherapists), and percentage of inhabitants who do not own their main property (Non-Owner). It was positively correlated with spatial accessibility to home-visiting nurses (APL_Nurses).

**Table 4:** Comparison of the OLS, SAR and GWR models for LOS in MCO and SSR facilities

Predictor variables	OLS		SAR		GWR	
	Coefficients	Std Error	Coefficients	Std Error	Coefficients	Std Error(mean)
LOS_MCO						
ISA_MCO	− 0.161***	0.037	0.015	0.024	[− 55.959; 8.438]	0.037
APL_GPs	− 1.065**	0.097	− 0.348***	0.061	[− 37.882; 37.084]	0.097
APL_Nurses	0.302***	0.096	0.005	0.065	[− 39.567; 78.291]	0.097
APL_Physiotherapists	− 1.518***	0.165	− 0.953***	0.108	[− 26.147; 33.852]	0.164
Non− Owner	− 1.395***	0.117	− 0.244***	0.076	[− 4.631; 2.412]	0.116
Precarious	− 0.507	0.403	− 0.415	0.253	[− 13.835; 10.757]	0.39
Spatial parameters						
Spatial Lag Coefficient ρ			0.767***			
Bandwidth GWR					Adaptive, 0.38% nearest neighbouring IRIS	
Model assessment						
Adjusted R^2^	0.508		0.775		0.955	
AIC	3285.273		2238.6		539.139	
Moran’s I residuals	0.609***		0.031*		0.086***	
LOS_SSR						
ISA_SSR	0.245***	0.036	0.131	0.023	[− 7.808; 4.756]	4.241
APL_GPs	0.332***	0.096	0.034	0.06	[–33.213; 57.124]	0.931
APL_Nurses	− 1.367***	0.09	− 0.423***	0.063	[− 28.11; 25.561]	1.124
APL_Physiotherapists	− 1.679***	0.165	− 1.048***	0.108	[− 20.186; 18.678]	0.811
Non− Owner	− 1.209***	0.114	− 0.169***	0.073	[− 4.631; 2.412]	1.133
Precarious	− 0.491	0.384	− 0.452	0.241	[− 7.075; 9.112]	0.473
Spatial parameters						
Spatial Lag Coefficient ρ			0.764***			
Bandwidth GWR					Adaptive, 0.52% nearest neighbouring IRIS	
Model assessment					0.931	
Adjusted R^2^	0.485		0.769		939.464	
AIC	3255.05		2181.912		0.131***	
Moran’s I residuals	0.611***		0.032*			

** p < 0.01; *** p < 0.001; Std Error: Standard deviation error

Compared with the results obtained with OLS, in the SAR model that controls the global spatial effect, the number of variables significantly associated with LOS decreased from five to three (APL_GPs, APL_Physiotherapists and Non-Owner). These three independent variables remained negatively associated with LOS. The absolute value of the coefficients and standard errors of each variable decreased. Furthermore, the magnitude of the regression coefficients suggested that spatial accessibility to physiotherapists had a greater global effect on LOS than spatial accessibility to GPs. Other factors, such as spatial accessibility to MCO facilities and to home-visiting nurses and percentage of inhabitants with a precarious employment (Precarious), did not significantly affect LOS in the SAR model.

By incorporating the mean LOS in neighbouring IRIS units into the equation, SAR took into account an estimate of the potential effect of neighbouring IRIS on LOS. For example, the spatial lag coefficient $\uprho$ for MCO was 0.767 (p < 0.001) (Table 4). This means that for each 1 unit increase in LOS at a given IRIS, their neighbours’ LOS increased by 0.767 units. However, this spatial effect was assumed to be the same across all IRIS units in the study area.

GWR was developed to highlight local effects. It adds additional information by estimating the regression coefficients for each area unit. Unlike OLS and SAR that give a global estimate, GWR produces local regression coefficients to highlight non-stationarity. The regression coefficients of GWR (Table 4) varied in the study area. The sign of the coefficients for different IRIS changed for all independent variables. This indicated that the effects of independent variables on LOS were not constant throughout the studied region and differed among IRIS units. For example, the local regression coefficients for APL_GPs varied from − 37.882 to 37.084, showing that the influence of spatial accessibility to GPs on LOS had various magnitudes and even different directions.

### Spatially varying parameters given by GWR

**Fig. 3:**
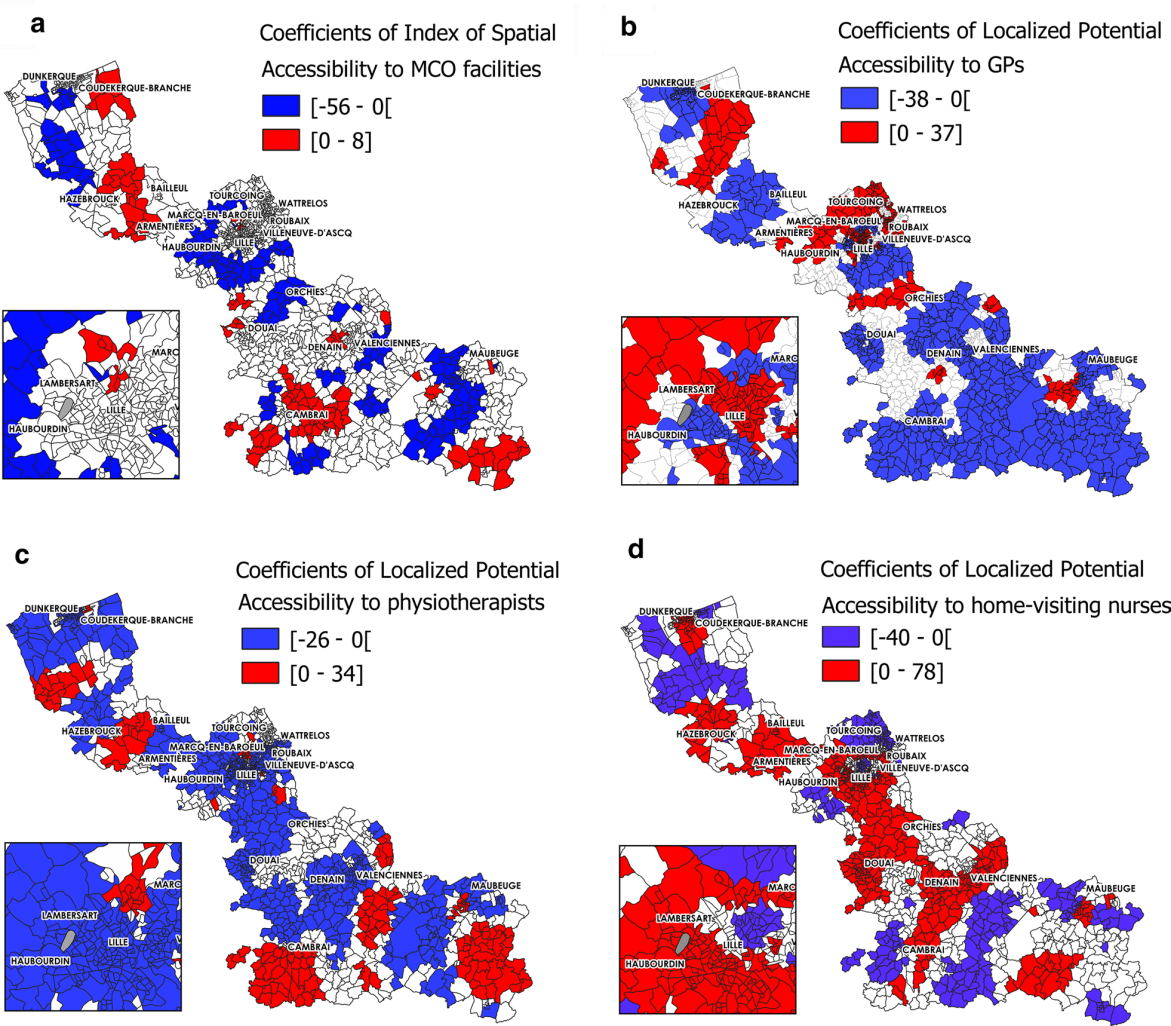
Significant GWR coefficient estimates $(\alpha$-risk level of 0.05), local R^2^ and bandwidth (MCO facilities) (part 1). Coefficients for **a** ISA to MCO, **b** APL to GPs, **c** APL to physiotherapists, **d** APL to home-visiting nurses

**Fig. 4:**
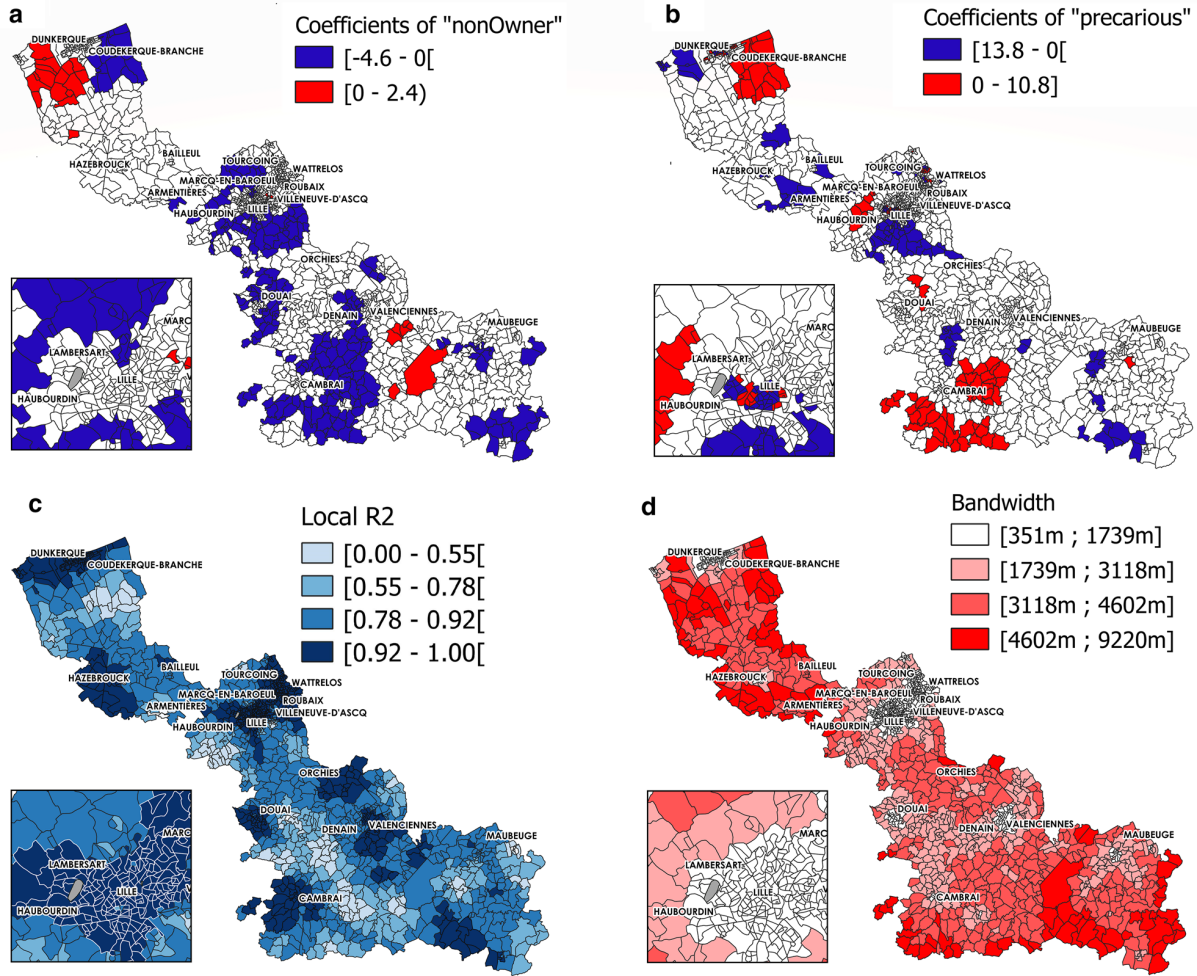
Significant GWR coefficient estimates $(\alpha$-risk level of 0.05), local R^2^ and bandwidth (MCO facilities) (part 2). Coefficients for **a** Non-Owners, and **b** Precarious, **c** Local R^2^. **d** Bandwidth

From the GWR model, local $\beta s$ were estimated for each independent variable. Figures 3 and 4 shows the spatial variations of the regression coefficients $\beta$.

The results of the GWR model showed the spatial heterogeneity of the associations with LOS. For instance, the spatial variation in the association between LOS in MCO and spatial accessibility to GPs was apparent (Fig. 3b), with 38.11% and 23.11% of IRIS units having a significant negative (mainly in the south or around Dunkerque and Bailleul) and positive association (mainly in urban areas in the centre, around Lille, Roubaix and Valenciennes), respectively. The associations between LOS in MCO and spatial accessibility to physiotherapists (Fig. 3c) displayed a completely different spatial distribution. For 55.35% of IRIS units, this relationship was negative, as expected (i.e. when spatial accessibility to physiotherapists increased, LOS number decreased); however, for 13.97% of IRIS units, particularly in the urban areas of the centre and in the southern part, the relationship was positive. These findings indicate that these two indexes of spatial accessibility to primary care services had completely different local influence on LOS. The local adjusted R^2^ values varied across the studied region, and were higher in the urban areas in the centre (Fig. 4c). The adaptive bandwidth used in the GWR model to take into account the hugely variable size of the geographical units was smaller for small IRIS (Fig. 4d).

Coloured areas show areas where the indicated parameter is significantly associated with length of hospital stay (LOS). Red, positive correlation; blue, negative correlation.

Only $\beta$ coefficient values that were significant at an α-risk level of 0.05 are presented. To be continued in Fig. 4.

Coloured areas show areas where the indicated parameter is significantly associated with length of hospital stay (LOS). Red, positive correlation; blue, negative correlation.

Only $\beta$ coefficient values that were significant at an α-risk level of 0.05 are presented.

#### Spatial model assessment & comparison

##### AIC and adjusted R^2^

The performance of each model (OLS, SAR, and GWR) used in this study was assessed by measuring the coefficient of determination (adjusted R^2^) and AIC. The very high R^2^ for the GWR model (0.955 for MCO and 0.931 for SSR) indicated that in this model, the independent variables explained the largest part of the variance of the dependent variable compared with the other two. R^2^ was 0.775 and 0.508 for MCO, and 0.769 and 0.485 for SSR, with the SAR and OLS models, respectively. The AIC value for the GWR model was 539 for MCO, lower than the AIC values for the SAR (2238) and OLS (3285) models.

##### Monte-Carlo test for GWR

The Monte-Carlo test was used to verify the significant spatial variability of the GWR model individual coefficients. The p-values < 0.05 of the independent variables, except for Non-Owner and Precarious, confirmed the significant spatial variation in the local coefficient estimates for all four indexes of spatial accessibility to primary and secondary care services. This result confirmed that most of the independent variables varied significantly across space (for both MCO and SSR), and stressed the importance of using models that take into account the spatial non-stationarity in our study.

##### Residual analysis using Moran’s I and local indicators of spatial association

**Fig. 5:**
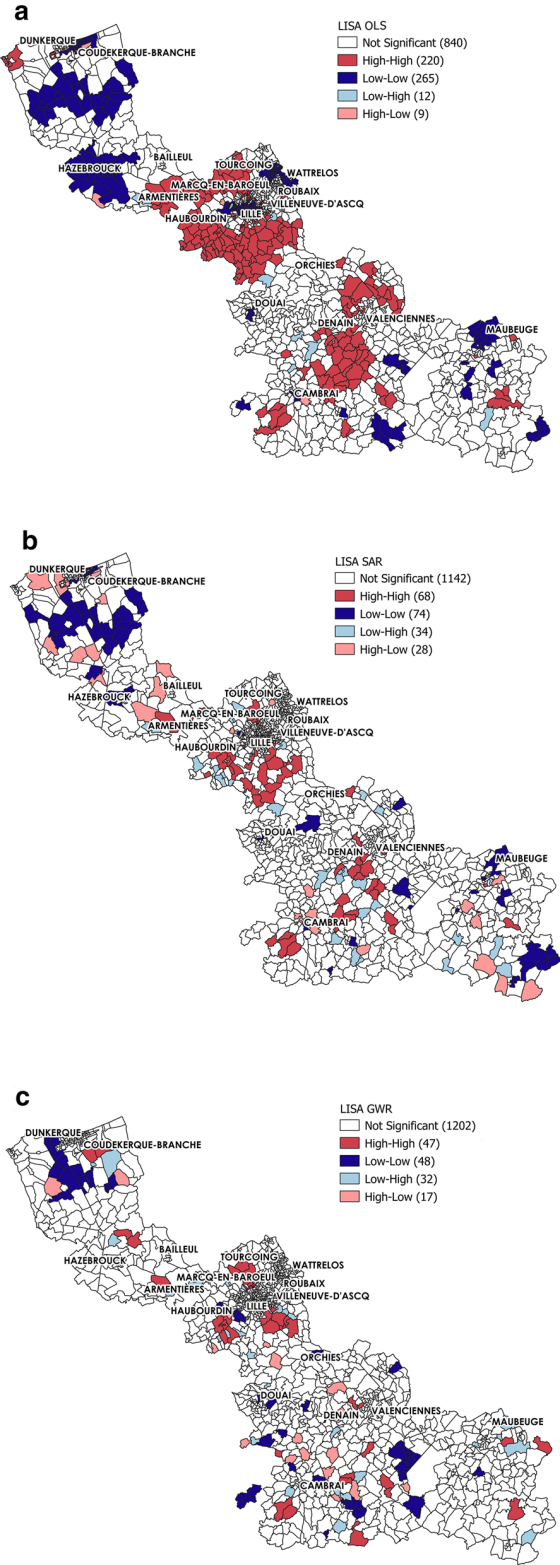
LISA map of OLS, SAR and GWR Model Residuals (MCO analysis). The red areas indicate areas with high LOS residuals surrounded by other areas with high LOS residuals (HH). The blue areas indicate areas of low LOS residuals surrounded by other areas with low LOS residuals (LL). The light blue areas indicate areas with low LOS residuals surrounded by areas with high LOS residuals (LH). The pink areas indicate areas with high LOS residuals surrounded by areas of low LOS residuals (HL). White areas, no significant spatial clustering. p < 0.01

The LISA indicator maps showed that in the OLS, SAR, and GWR models (for MCO), spatial clustering of LOS residuals occurred in statistically significant patterns (Fig. 5). In the OLS model, residuals exhibited spatial autocorrelation in many areas across the studied region (Fig. 5a). Autocorrelation clusters were smaller with the SAR model (Fig. 5b). The spatial autocorrelation of residuals was reduced and residuals were spatially clustered only in 204 IRIS units (68HH, 74LL, 34LH, and 28HL). The number of spatially associated clusters was lowest in the GWR model (Fig. 5c): only 126 IRIS units (47HH, 48LL 32LH, and 28HL).

Moreover, the Moran’s I values for the residuals varied similarly in the OLS, SAR and GWR models (Table 4). Moran’s I value significantly decreased between the OLS and GWR models (0.609 to 0.086). The lowest Moran’s I (0.031) was obtained with the SAR model. This value was very close to 0, suggesting an absence of spatial autocorrelation in LOS residuals.

## Discussion

To our knowledge, this is the first study that investigated the efficiency of fine scale and spatial regression in modelling associations between healthcare service spatial accessibility and length of stay. Different spatial scales and regression models were examined.

### Comparison with the international literature

Previous studies explored the association between healthcare spatial accessibility and utilization by different perspectives. In most cases, researchers focused on the spatial accessibility and utilization of only one type of healthcare service (primary or secondary). For instance, Jones et al. found that due to longer travel time, patients living further away from the provider were less likely to make doctor’s appointments compared with those living closer [113]. Similarly, Arcury et al. [107] demonstrated that greater distance resulted in fewer regular check-up visits. Kim et al. found that a reduction of the travel time to hospital increases their utilization by patients with chronic obstructive pulmonary disease [74]. Another study did not observe any significant association between spatial accessibility by car or public transport and utilization of gynaecologists and GPs [29]. In these studies, the interaction between ambulatory/home care and inpatient hospital care was not considered. Other authors studied the relationship between primary care spatial accessibility and inpatient hospital care utilization. Bindman et al. [37] and Daly et al. [41] demonstrated significant associations between preventable hospitalizations and access to primary care. Several groups examined the role of primary care spatial accessibility in inpatient care/emergency department utilization. Specifically, Bindman et al. showed that access to primary care was inversely associated with the hospitalization rates for five chronic medical conditions. Daly et al., Fishman et al., and Huang et al. found that the odds of preventable emergency department use are higher in patients living in medically underserved areas. Kjekshus et al. analysed the interaction between primary and inpatient hospital healthcare services in Norway and the effect on LOS. They highlighted that LOS was influenced by the primary healthcare provider capacity and also by the percentage of elderly in the hospital catchment area, type of patients, coordination procedure, and intrinsic features of the hospital [47]. In many of these studies, hospital care utilization was measured using the admission rate or LOS. The evaluation of spatial accessibility was based on the self-rated access level [37], network distance/travel time [29, 74, 113, 114], or the E2SFCA method that takes into account both healthcare density and proximity [39, 41, 55]. The statistical methods used to investigate these associations were based on classical regression models [29, 37, 39, 47, 55, 74], or global spatial lag models [41].

### Interpreting the study results at the global scale

The main strengths of our study are the concomitant assessment of 1) primary care spatial accessibility, 2) inpatient hospital spatial accessibility, and 3) hospital care utilization. Moreover, the OLS, SAR and GWR models allowed obtaining results both at the global and local scale. At the global level, the analysis revealed a significant and negative association between LOS of elderly people (≥ 75 years) and spatial accessibility to GPs and physiotherapists. In other words, better spatial accessibility to these two primary care services corresponded to shorter hospital stays. This finding could be explained by the hypothesis that inpatient hospital care facilities and primary care services may interact in a complementary way. Specifically, in areas with better spatial accessibility to primary care, hospital stays could be shorter thank to the presence of effective primary care services (e.g. outpatient care and neighbourhood healthcare services). This result appears coherent with the findings of previous studies [115–117]. As mentioned by Kjekshus et al., in most countries, after hospital treatment, a patient should be followed by primary healthcare services, and the ability of the primary healthcare services to take care of such patients is believed to have a significant impact on LOS, particularly for elderly people in whom the recovery period is often longer [47].

### Local scale results: Efficiency of the geospatial modelling approach

As no previous study compared the efficiency of traditional regression analyses with a geospatial approach for modelling the relationship of potential and realized healthcare access, this study compared the results obtained with the OLS, SAR, and GWR models. The main weakness of the OLS approach is that it ignores spatial non-stationarity and spatial autocorrelation. SAR, which addressed the spatial autocorrelation issue, gave more robust results than OLS. However, one limitation of the SAR model is that all observations share a common spatial effect ρ. Yet, spatial variation in geographic data is seldom constant across a study area. This issue was addressed by the GWR model that can highlight regional patterns of spatially varying parameter estimates, thus revealing additional insights at local scales. This allowed determining whether the relationship between potential and realized access was intrinsically different across space. Whatever the model, our study found a negative association between LOS and access to GPs and to physiotherapists at the global scale. However, the GWR model revealed that for some areas (i.e. local analysis), this relationship was positive, and that the distribution of areas positively or negatively correlated with LOS varied when considering the access to GPs and to physiotherapists. Only the GWR model allowed highlighting this level of information.

Statistically speaking, SAR and GWR improved the model performance. However, GWR ranked first (highest adjusted R^2^ and lowest AIC). After applying spatial models, the spatial autocorrelation of residuals was significantly reduced in the SAR model. This reduction was lower in the GWR model, as indicated by its Moran’s I values.

In terms of public health, the ability to identify how these relationships vary in space could bring important information to engage discussions about healthcare public policy, hospital decision-making and healthcare resource allocation: 1) for areas where primary care spatial accessibility is negatively correlated with LOS, it would be important to make sure that there is already a coordinated approach between primary and secondary care services, and to allocate more resources to community care (especially specific healthcare professional types in function of the population needs); 2) for areas with high spatial accessibility to primary healthcare services and long LOS (i.e. positive correlation), it should be determined whether the primary services can properly follow patients after hospital discharge. Moreover, a consolidated approach should be developed to facilitate the care pathway coordination. The objective is to contribute to the sustainability of inpatient and outpatient care services, to complement inpatient hospital care with primary care, and to increase healthcare efficiency.

Finally, although spatial analyses provide a tool for exploring the impact of potential accessibility to primary and secondary healthcare services on LOS, our preliminary quantitative results should be supplemented by a qualitative approach to better understand them. Future investigative fieldwork and analyses of the spatial accessibility effect on LOS should include different dimensions related to care pathway coordination, such as patient and health professional types and their behaviours, hospital characteristics, procedure performed, financial issues and service quality. These dimensions should provide a more comprehensive analysis of healthcare service access and utilization.

### Contributions and limitations

Our study investigated the impact of spatial accessibility to hospital and primary healthcare services and socio-economic factors on LOS. The use of multiple spatial scales and regression models allowed assessing the spatial heterogeneity and spatial dependence of different factors. Moreover, it assessed the efficiency of fine scale and spatial regression model analysis. However, some limitations need to be acknowledged. First, we chose the LOS to describe healthcare utilization. Besides spatial accessibility, this indicator could be influenced by other factors, such as disease severity, disease type, treatments reimbursed and chosen by the patients. These factors were not included in the present case study on the relationship between LOS and spatial accessibility. Then, in our potential and realized access framework, we included hospital and primary healthcare service spatial accessibility, and a hospital service utilization indicator. We did not have information about primary care service utilization. The socio-economic variables involved in this study were limited in number, and therefore their analysis was preliminary. As more data become available, more influential factors could be added to the spatial models to obtain a better explanation of the relationships between potential and realized healthcare access. In addition, as our models are based on E2SFCA metrics and data aggregated at the FGC or IRIS level, there may be an ecological fallacy and data may not reflect the associations at the individual level within the territory. Furthermore, some data were only available at the FGC level and were disaggregated to the IRIS level in a homogeneous way. For future studies, we want to construct LOS and APL indicators at a finer scale using more sophisticated disaggregation techniques.

## Conclusion

Examining the association of LOS with primary and secondary healthcare service spatial accessibility provides information that may be exploited for public health planning. The comparison of the OLS, SAR and GWR models showed that spatial regressions are useful for these analysis. GWR performed better and could provide additional insights by revealing the hidden spatial distribution patterns of coefficient estimates and their statistical significance.

The finding of “spatially varying relationships” between healthcare spatial accessibility and LOS is important because it can help to better understand the complicated links between healthcare spatial accessibility and utilization, and between primary and secondary healthcare. It should be noted that the GWR method only revealed the spatially varying patterns, and additional research is needed to obtain more insights into the causal effects of primary and secondary healthcare service spatial accessibility and length of stay.

## Data Availability

All data generated or analyzed during this study are included in this published article. If readers need supplementary information, they can contact me (fei.gao@ehesp.fr).

## Abbreviations

APL: Localized Potential Accessibility
E2SFCA: Enhanced two-Step Floating Catchment Area
FINESS: National File on Health and Social Institutions
FGC: French Geographic Code unit
ISA: Spatial Accessibility Index
MCO: Medical, Surgical and Obstetrics
SAE: Annual Statistical Survey of Healthcare Facilities
SSR: Postoperative and Rehabilitation Care
LOS: Length Of Stay
